# Drug-induced thrombotic microangiopathy: An updated review of causative drugs, pathophysiology, and management

**DOI:** 10.3389/fphar.2022.1088031

**Published:** 2023-01-09

**Authors:** Tommaso Mazzierli, Federica Allegretta, Enrico Maffini, Marco Allinovi

**Affiliations:** ^1^ Nephrology, Dialysis and Transplantation Unit, Careggi University Hospital, Florence, Italy; ^2^ Department of Biomedical Experimental and Clinical Sciences “Mario Serio”, University of Florence, Florence, Italy; ^3^ Department of Hematology, IRCCS Azienda Ospedaliero-Universitaria di Bologna, Bologna, Italy

**Keywords:** thrombotic microangiopathy, hemolytic-uremic syndrome, thrombotic thrombocytopenic purpura, drug-induced, toxicity, nephrotoxicity

## Abstract

Drug-induced thrombotic microangiopathy (DITMA) represents 10%–13% of all thrombotic microangiopathy (TMA) cases and about 20%–30% of secondary TMAs, just behind pregnancy-related and infection-related forms. Although the list of drugs potentially involved as causative for TMA are rapidly increasing, the scientific literature on DITMA is quite scarce (mostly as individual case reports or little case series), leading to poor knowledge of pathophysiological mechanisms and clinical management. In this review, we focused on these critical aspects regarding DITMA. We provided an updated list of TMA-associated drugs that we selected from a scientific literature review, including only those drugs with a definite or probable causal association with TMA. The list of drugs is heterogeneous and could help physicians from several different areas to be familiar with DITMA. We describe the clinical features of DITMA, presenting the full spectrum of clinical manifestations, from systemic to kidney-limited forms. We also analyze the association between signs/symptoms (i.e., malignant hypertension, thrombocytopenia) and specific DITMA causative drugs (i.e., interferon, ticlopidine). We highlighted their multiple different pathophysiological mechanisms, being frequently classified as immune-mediated (idiosyncratic) and dose-related/toxic. In particular, to clarify the role of the complement system and genetic deregulation of the related genes, we conducted a revision of the scientific literature searching for DITMA cases who underwent renal biopsy and/or genetic analysis for complement genes. We identified a complement deposition in renal biopsies in half of the patients (37/66; 57%), with some drugs associated with major deposits (i.e., gemcitabine and ramucirumab), particularly in capillary vessels (24/27; 88%), and other with absent deposits (tyrosine kinase inhibitors and intraocular anti-VEGF). We also found out that, differently from other secondary TMAs (such as pregnancy-related-TMA and malignant hypertension TMA), complement genetic pathological mutations are rarely involved in DITMA (2/122, 1.6%). These data suggest a variable non-genetic complement hyperactivation in DITMA, which probably depends on the causative drug involved. Finally, based on recent literature data, we proposed a treatment approach for DITMA, highlighting the importance of drug withdrawal and the role of therapeutic plasma-exchange (TPE), rituximab, and anti-complementary therapy.

## 1 Introduction

Thrombotic microangiopathy (TMA) is a pathological syndrome characterized by microangiopathic hemolytic anemia (MAHA), thrombocytopenia, and organ damage, with predominant involvement of the kidneys and/or the brain (Fakhouri et al., 2017).

Attempts at classification of different TMA forms are complex and in continuous evolution. Several approaches have been proposed, each taking into account different aspects, such as genetic involvement and secondary causative agents (Brocklebank et al., 2018). The key feature of the different TMA forms, both primary and secondary, is endothelial damage. In drug-induced TMA (DITMA), the mechanism of endothelial damage induced by different drugs is heterogeneous and it can involve direct endothelial damage, alteration of ADAMTS-13 activity or deregulation of different endothelial biological pathways (such as VEGF and NF-kB pathways) (Goldberg et al., 2010; Mathew et al., 2016; Chatzikonstantinou et al., 2020).

Secondary TMAs account for the vast majority of TMAs (80%–90%) (Kang et al., 2017; Bayer et al., 2019). DITMA accounts for 10%–13% of all TMA cases and about 20%–30% of the secondary TMAs, representing the third most frequent cause, after pregnancy-related and infection-related forms (Bayer et al., 2019; Beloncle et al., 2021). Although the list of drugs potentially involved as causative for TMA are rapidly increasing, the scientific literature on DITMA is quite scarce, especially if compared to surveys on primary TMAs [e.g., atypical hemolytic-uremic syndrome (aHUS) and thrombotic thrombocytopenic purpura (TTP)], which are deemed to be rarer clinical entities. The real incidence of DITMA remains undetermined but is probably underestimated.

In this review, we focused on different critical aspects regarding DITMA: the underlying pathophysiological mechanism, the dysregulation of the complement system (and the potential genetic predisposition), and different treatments. Our proposal opens for future developments in DITMA study and treatment.

## 2 Methods

A cause-effect relationship between a given drug and TMA development is not always easy to prove because of possible confounding factors such as ongoing infections, comorbid conditions and presence of other DITMA-associated drugs, frequently coexisting in a single patient.

Criteria and levels of evidence for an association of a single drug with TMA were defined using criteria by Al-Nouri et al. (2015), previously adapted from drug-induced thrombocytopenia (George et al., 1998). The criteria differ whether the etiopathogenic mechanism is immune or toxic.

Therefore a specific drug is identified as potential causative agent of TMA, with a definite or probable causal association, with a toxic mechanism in relation to: i) association of drug exposure with the presence of clinical or pathologic criteria for TMA; ii) exclusion of clinically apparent causes of clinical/pathologic criteria other than TMA and exclusion of causes of TMA other than drug toxicity; also the suspected drug was the only drug taken or other drugs were continued or restarted; iii) resolution or improvement of TMA when the suspected drug was stopped or the dose is reduced (even if kidney injury may persist).

The criteria to establish the presence of an immune-mediated DITMA, with a definite or probable causal association between drug exposure and TMA, are: i) Clinical or pathologic diagnostic criteria for TMA were present; ii) clinically apparent causes of clinical/pathologic criteria other than TMA, and causes of TMA other than drug toxicity were excluded and the suspected drug was the only drug taken or other drugs were continued or restarted and if the suspected drug had been taken daily, systemic symptoms occurred within 21 days of starting the drug, or if the suspected drug had been taken intermittently, systemic symptoms began suddenly, within 24 h of drug exposure; iii) previous or subsequent drug exposure associated with systemic symptoms or TMA; iv) drug-dependent antibodies were documented, reactive with platelets or other cells. These criteria are important both in research and clinical activity, to evaluate TMA cases correctly and give drugs a proper role in a clinical setting of TMA.

A “definite” causal association between drug exposure and TMA was established by either clinical criteria (repeated drug exposures associated with recurrent TMA episodes) or by laboratory criteria (documentation of drug-dependent antibodies to platelets, neutrophils, complement factors, endothelial cells), together with a temporal criteria (therapy with the candidate drug preceded TMA) and the exclusion of etiologies of TMA other than drug toxicity.

We searched on PubMed to identify publications discussing DITMA, using medical subject heading terms: “thrombotic microangiopathy”, “haemolytic uremic syndrome”, “thrombotic thrombocytopenic purpura”. We updated the list of TMA-associated drugs matching the above listed criteria, including only those drugs with a definite or probable causal association with TMA described in papers published from January 2017 to April 2022 and not already reported by (Al-Nouri et al., 2015; Saleem et al., 2018) (Table 1). The association level and the references which support the causative relationship between new drugs and DITMA are reported in Supplementary Table S1–S17.

**TABLE 1:** Drugs reported to have a probable or definite association with TMA.

Chemotherapeutic drugs	Targeted cancer drugs	Immunosuppressive drugs	Antibiotics and antivirals	Other drugs
Daunorubicin	Alemtuzumab	Adalimumab	Ciprofloxacin	Onasemnogene abeparvovec
Docetaxel	Moxetumomab pasudotox	Certolizumab pegol	Levofloxacin	Clopidogrel
Gemcitabine	Imatinib mesylate	Cyclosporine A	Metronidazole	Bupropion
Lomustine	Lenvatinib	Tacrolimus	Penicillin	Valproic acid
Mitomycin C	Nintedanib	Interferon (alpha, beta,polycarboxylate)	Rifampicin	Estrogen/Progesterone
Oxaliplatin	Palbociclib	Leflunomide	Sulfisoxazole	Hydroxychloroquine
Pegylated liposomaldoxorubicin	Pazopanib	Muromonab-CD3 (OKT3)	Trimethoprim-sulfamethoxazole	Oxycodone hydrochloride ER
Pentostatin	Regorafenib	Sirolimus	Famciclovir	Oxymorphone ER
Tamoxifen	Sunitinib	Everolimus	Valacyclovir	Quetiapine
Trastuzumab	Bevacizumab			Quinine
Vincristine	Ramucirumab			Simvastatin
Lomustine				
Bortezomib				Illicit drugs and toxic substances
Carfilzomib				Cocaine
Ixazomib				Ecstasy
				Trielina

Legend: Definite association; Probable association.

To investigate a potential complement involvement in DITMA cases, we conducted a revision of the scientific literature on PubMed searching for case reports or case series on patients with DITMA who underwent to renal biopsy and reporting immunofluorescence (IF) staining for C3, C4 and C1q. All cases are summarized in Table 2 and referenced in Supplementary Material S18–S53.

**TABLE 2:** Complement serum consumption and complement activation in renal biopsies of patients with DITMA.

Author	Patient (age)	Causative drug	Disease	Serological markers	Renal biopsy (immunofluorescence staining)	Complement involvement (0 = No, 1 = only blood, 2 = only biopsy, 3 = both)
Li Cavoli [2011][S18]	F, 36	IFN-β	Multiple sclerosis	NA	chronic TMA	2
					IF: IgM and C1q ++,mesangial	
Broughton [2011][S19]	F, 53	IFN-β	Multiple sclerosis	normal C3 and C4 levels	subacute TMA	2
					IF: C3++	
Olea [2012][S20]	F, 37	IFN-β	Multiple sclerosis	NA	chronic TMA	0
					IF: negative	
Nerrant [2013][S21]	F, 38	IFN-β	Multiple sclerosis	normal C3 and C4 levels	acute TMA	0
				normal ADAMTS13	IF: IgM	
Piccoli [2016][S22]	M, 31	IFN-β	Multiple sclerosis	Normal C3 and C4 levels	acute TMA, collapsing glomerulopathy	0
					IF: negative	
Allinovi [2017][S23]	M, 35	IFN-β	Multiple sclerosis	Low C3 levels and normal C4 levels; normal ADAMTS13	acute TMA	3
					IF:C3++,subendothelial and capillary wall	
Gianassi [2019][S24]	F, 55	IFN-β	Multiple sclerosis	low C3 levels and normal C4 level; normal ADAMTS13	acute TMA	3
					IF: C3+, capillary wall	
Parisi [2020][S25]	M, 39	IFN-β	Multiple sclerosis	normal C3/C4 levels	acute TMA	0
				normal ADAMTS13	IF: IgG +	
Allinovi [2021][S26]	M, 38	IFN-β	Multiple sclerosis	low C3 level and normal C4 levels	TMA	3
					IF: C3++, capillary wall	
Murugapandian [2015][S27]	F, 74	Gemcitabine	Ovarian carcinoma	Normal C3 and C4 levels	Acute TMA	0
					IF: negative	
Krishnappa [2018][S28]	F, 69	Gemcitabine	Pancreatic adenocarcinoma	Low C3 and C4 levels; normal ADAMTS13	Acute TMA	3
					IF:C3++, capillary wall	
Burns [2020][S29]	M, 39	Gemcitabine	Pancreatic adenocarcinoma	Normal C3 and C4 levels; normal ADAMTS13	Acute TMA	2
					IF:C3+++,C1q+ in capillary wall	
Grall [2021][S30]	12 patients	Gemcitabine	Pancreas, ovarian, lung and uterine adenocarcinoma	8 patients: normal C3 and C4 levels	TMA IF: all patients C5b9 +++ in tubule, glomerulus and capillary wall	2 8)
				4 patients: low C3		3 4)
				All patients: normal ADAMTS13		
Etta [2020][S31]	M, 44	Clopidogrel	Unstable angina	Normal C3 and C4 levels	Acute TMA	0
					IF: negative	
Mizuno [2021][S32]	M, 75	Bortezomib	Multiple myeloma	Normal C3 and C4 levels; normal ADAMTS13	MPGN and TMA	0
					IF: IgG +++	
Hobeika [2014][S33]	M, 62	Carfilzomib	Multiple myeloma	Normal C3 and C4; normal ADAMTS-13	TMA and podocytopathy	2
					IF: C3, C1q +++, IgM and fibrin in interlobular artery	
Yamada [2019][S34]	F, 77	Ramucirumab	Colic adenocarcinoma	Normal C3 and C4	Acute TMA	2
					IF: C1q and IgM++ in mesangium and capillillary wall	
Ozawa [2019][S35]	9 patients	Ramucirumab and bevacizumab	Colon, rectum, lung and brain cancer	NA	Acute TMA	2 9)
					IF: all patients C4+/++, 4 patients C3+, 5 patients C3 negative	
Nakano [2021][S36]	M, 71	Ramucirumab	Cecal cancer	NA	TMA and collapsing glomerulopathy	2
					IF: C3 ++, C4, C1q, IgA, IgM and IgG + in mesangium and capillary wall	
Pellé [2011][S37]	M, 77	IO anti-VEGF	Age-related macular degeneration	Normal C3 and C4; normal ADAMTS-13	TMA	0
					IF: negative	
Touzani [2019][S38]	M, 72	IO anti-VEGF	Glaucoma	Normal C3 and C4; normal ADAMTS-13	TMA	2
					IF:C3++ in capillary wall	
Hanna [2020][S39]	M, 59	IO anti-VEGF	Diabetic retinopathy	NA	Chronic TMA IF: negative	0
	F, 43		Diabetic retinopathy	NA	Acute TMA IF: negative	0
	M, 77		Diabetic retinopathy	NA	Chronic TMA IF: negative	0
Miller [2019][S40]	F, 70	DCR-MYC (short interfering RNA)	Breast adenoid cystic carcinoma	NA	Chronic TMA	0
					IF: negative	
Eremina [2008][S41]	6 patients	Bevacizumab	Hepatic, lung carcinoma, pancreatic and ovarian cancer	NA	TMA	0
					IF: 4 negative and 2 IgA++ patients	
Roncone [2007][S42]	M, 59	Bevacizumab	Renal cell carcinoma	NA	Chronic TMA	2
					IF: IgG, IgA, IgM, C3 ++ in mesangium and capillary wall	
Toriu [2019][S43]	M, 88	Bevacizumab	Colorectal cancer	Normal C3 and C4; normal ADAMTS-13	Acute TMA	2
					IF: C3 and IgM ++ in mesangial and capillary wall	
Morimoto [2021][S44]	M, 68	Bevacizumab	Ovarian carcinoma	Normal C3 and C4	TMA	2
					IF: IgG, IgA, IgM, C3, C4, and C1q ++ in subendothelium and mesangium	
Yilmaz [2016][S45]	M, 15	Polychemotherapy	Osteosarcoma	Normal C3 and C4; normal ADAMTS-13	Acute TMA	2
					IF: IgG, IgA, IgM, C3, C4, and C1q ++	
Morita [2022][S46]	M, 79	Imatinib	Chronic myeloid leukemia	NA	Chronic TMA and podocytopathy	0
					IF: negative	
Patel [2008][S47]	5 patients	Sunitinib	GIST, epithelioid hemangio-endothelioma and teratocarcinosarcoma	NA	TMA	0
					IF: negative	
Bollée [2009][S48]	F, 44	Sunitinib	Malignant skin hydro adenoma	normal C3 and low C4; normal ADAMTS-13	Acute TMA	3
					IF: IgM, IgG, IgA, C3 and C1q ++ in mesangium and capillary wall	
Jha [2013][S49]	M, 60	Sunitinib	Clear-renal cell carcinoma	NA	Acute TMA	0
					IF: IgM+	
Nieto-Rios [2021][S50]	M, 74	Sunitinib	GIST	Normal ADAMTS-13	Chronic TMA and podocytopathy	0
					IF: negative	
Hyogo [2018] [S51]	F, 70	Lenvatinib	Papillary thyroid carcinoma	NA	Chronic TMA	0
					IF: negative	
Hasegawa [2020][S52]	M, 69	Nintenatib	Pulmonary idiopathic fibrosis	Normal C3 and C4; normal ADAMTS-13	Acute TMA	0
					IF: IgM+	
Maruyama [2018][S53]	M, 31	Pazopanib	Rhabdomyosarcoma	NA	Acute TMA	2
					IF: IgA, IgM, C3, C4, C1q ++ along the glomerular basement mebrane	

Legend: IF, immunofluorescence staining; IFN-β, interferon beta; IO, intraocular; F, female; M, male; MPGN, membranoprolipherative glomerulonephritis; NA, not available; TMA, thrombotic microangiopathy.

Moreover, to investigate a potential underlying genetic predisposition, we searched for DITMA cases describing at least two patients (no single case reports) and testing for the complement gene panel (CFH, CFHR, C3, CFI, CFB, MCP and THBD). Re-evaluation of all the identified variants was based on ClinVar. Variants were reported with the use of specific standard terminology - “pathogenic”, “likely pathogenic”, “uncertain significance”, “likely benign” and “benign”- according to the ACMG guidelines (Richards et al., 2015). The results are shown in Table 3 and the references are listed in Supplementary Material S54–S64.

**TABLE 3:** The role of causative mutation in complement factors in patients affected by DITMA.

Author, year	Number of patients	Type of DITMA	Number of patients with an underlying causative mutation in complement factors	Number of patients with variants of undetermined significance or polymorphisms
Hunt, 2014 [S54]	5	IFN-beta	0/5	NA
Allinovi, 2021 [S55]	7	IFN-beta	1/7 heterozygous MCD (c.1058C > T)	3/7
				- heterozygous CFHR3/CFHR1 gene deletion 1)
				- homozygous for the risk haplotype CFH-H3 and a CFH (c.2650T > C) 1)
				- homozygous CFHR3/CFHR1 gene deletion and haplotype CFH-H3 1)
Dauvergne, 2021 [S56]	11	IFN-beta	0/11	1/11 two rare variants of undetermined significance in the CFI gene (not specified), associated with a homozygous deletion of CFHR1 1)
Le Clech, 2019 [S57]	32	Various drugs	1/32 heterozygous CFH (c.3596T>C)	1/32 heterozygous CFI (c.11T > A)
Schulte-Kemna, 2020 [S58]	2	Polychemotherapy	0/2	0/2
Grall, 2021 [S59]	9	Gemcitabine	0/9	0/9
Page, 2017 [S60]	2	Quinine	0/2	NA
Izzedine, 2013 [S61]	13	Sunitinib, VEGF trap, bevacizumab	0/13	0/13
Cavero, 2017 [S62]	15	Calcineurin inhibitors	0/15	5/15
				- heterozygous CFHR2 (Y264C) 2)
				- heterozygous MCP (c.-325 A>C) and heterozygousTHBD c.*36G>A) 1)
				- heterozygous THBD (c.*23_*40del) 1)
				- heterozygousTHBD: (c.*36G>A) 1)
Yui, 2016 [S63]	2	Proteasome inhibitor	0/2	0/2
Portuguese, 2020 [S64]	3	Carfilzomib	0/3	2/3 harbor heterozygous *CFHR3-CFHR1* deletion, no anti-CFH antibodies 2)
Gavriilaki, 2022 [S65]	17	Carfilzomib	0/17	NA
Chapin, 2013 [S66]	4	Thienopyridine	0/4	0/4
Total	122		2/122 (1.6%)	12/98 (12.2%)
				9/98 (9.1%) (without CFHR related mutations)

## 3 Etiopathogenic mechanisms and complement involvement in DITMA

The number of drugs associated with DITMA are rapidly increasing and present a heterogenous mechanism of action. Moreover, the role of the complement system in causing DITMA is not completely clear. In this paragraph, we updated the list of causative drugs in DITMA and we reviewed the etiopathogenic mechanisms underlying DITMA forms, with a major focus on complement overactivation and genetic deregulation in DITMA patients.

### 3.1 Heterogeneity of etiopathogenesis for DITMA classification: Description and limitations

Numerous drugs are described in scientific literature as causative of TMA, mostly as little case series, and the number is rapidly increasing (see Table 1). The drugs reported as causative of TMA are heterogeneous in terms of mechanism of action. Some drugs are more frequently used in clinical activity, as antibiotics or antiplatelet therapy, some others in more restricted contexts, such as IO-anti-VEGF. The heterogeneity of these causative drugs should require that physicians from several different areas are familiar with DITMA.

The most frequent drugs described as TMA causative agents are: calcineurin inhibitors (tacrolimus, cyclosporine, etc), thienopyridine (clopidogrel), anti-neoplastic drugs (gemcitabine, mitomycin, etc), VEGF inhibitors (bevacizumab) and VEGF receptor blockers (tyrosine kinase inhibitors, such as pazopanib and sunitinib), interferon (alpha, beta, gamma) and quinine. These groups of drugs account for more than 75% of all DITMA cases (Reese et al., 2015; Bayer et al., 2019).

**FIGURE 1:**
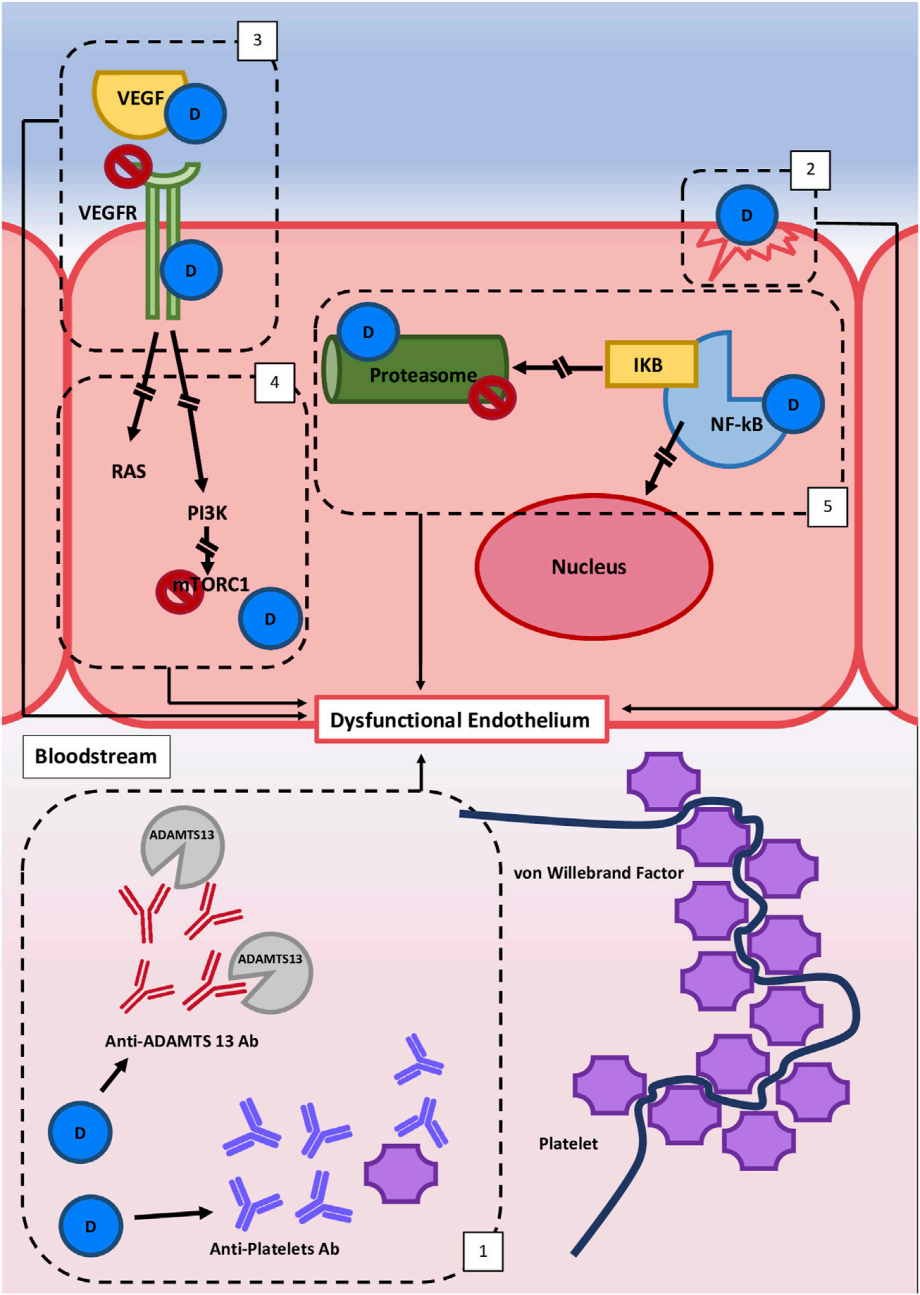
Hypothetical etiopathogenetic mechanisms involved in Drug-induced thrombotic microangiopathy (DITMA). Different etiopathogenetic mechanisms are involved in DITMAs and they all lead to endothelial dysfunction and/or platelet activation. These mechanisms are commonly distinguished in two subgroups. The first group is characterized by an immune-mediated reaction (1), related to abnormal susceptibility to a drug which leads to the production of reactive antibodies that interact with endothelium, platelets and/or circulating factors. The second group is bonded by a cumulative effect dose mechanism that leads to the interruption of different pathways variously involved in the maintenance of physiological endothelium homeostasis: (2) direct endothelial damage, (3) interference with signal protein and their receptors (e.g. VEGFR pathway), [4] interference with signal transducers (e.g. mTOR pathway), and (5) downregulation of transcription factors (e.g., NF-kB). Legend: D, drug; VEGF, vascular endothelial growth factor; VEGFR, vascular endothelial growth factor receptor; PI3K, Phosphoinositide 3-Kinase; mTORC1 = mTOR, Complex one; NF-KB, Nuclear Factor Kappa B; IKB, Inhibitor Kappa B; Ab, Antibody.

Considering that DITMA can be caused by different and heterogeneous mechanisms (Figure 1), the most frequent etiopathogenetic classification splits them in two subgroups: immune-mediated (idiosyncratic) and dose-related/toxic.

1) The idiosyncratic immune-mediated DITMA is related to abnormal susceptibility/hypersensitivity to a given drug. It is characterized by the production of reactive antibodies after the exposure to the putative drug. The antibodies interact with endothelium, platelets and/or circulating factors, favoring platelet-thrombi formation and endothelial damage, thus predisposing to the TMA development. The reported causative antibodies are mainly directed against platelets. Drug-dependent antiplatelet antibodies have been demonstrated for quinine (Al-Nouri et al., 2015; George et al., 2017), vancomycin and oxaliplatin (Reese et al., 2015). Moreover, other autoantibodies are reported but not clearly associated with drug exposure. For example, the presence of drug-dependent antibodies against ADAMTS-13 together with decreased anti-ADAMTS-13 activity are reported for interferon (Orvain et al., 2014; Gangaraju et al., 2017; Kundra and Wang, 2017) and for ticlopidine (Tsai et al., 2000; Zakarija et al., 2009; Bennett et al., 2013). Evaluating those case reports, the association between drug exposure, drug-dependent anti-ADAMTS-13 antibody and TMA did not fit with the temporal criteria adopted to define an immune-mediated DITMA. In conclusion, based on the literature data, it is possible to hypothesize the presence of drug-dependent anti-ADAMTS-13 antibodies as a causative mechanism underlying a few cases of immune-mediated DITMA (Figure 1), but further supporting evidence are needed to prove their causative role.
2) In the majority of DITMA cases, drugs cause TMA with a cumulative effect dose. The cumulative mechanism is related to progressive blockade of different pathways, mainly involved in maintenance of physiological endothelial homeostasis. Toxic effect on endothelial cells can be the result of:
  - direct endothelial damage with pro-thrombotic endothelial assessment, as caused by mitomycin (Sinitsky et al., 2020), gemcitabine (Zupancic et al., 2007), interferon (Kavanagh et al., 2016) and quinolones (Bezwada et al., 2008);
  - interference with signal protein and their receptor, such as VEGF-pathway, due to the activity of anti-VEGF monoclonal drugs (bevacizumab) or TK inhibitors (sunitinib, pazopanib *etc.*) (Estrada et al., 2019), that mainly affect kidney endothelial cells. Kidney susceptibility to VEGF blockade is related to VEGF pivotal role in maintaining the homeostasis of endothelial-podocyte complex (Eremina et al., 2008; Izzedine and Perazella, 2015);
  - interference with signal transducers, such as mTOR-pathway, with mTOR inhibitors (Everolimus) that interfere with endothelial cells and in particular with autophagy (a system of intracellular degradation that maintains the cell and organelle homeostasis) (Lenoir et al., 2015; Tagawa et al., 2016; Du et al., 2017; Zheng et al., 2020);
  - deregulation of transcription factors, such as NF-KB, with production of proinflammatory/prothrombotic mediators, augmentation of oxidative stress, increase of endothelin pathway, decrease in nitric oxide concentration and VEGF production with multi-organ diffuse endothelial damage. This pattern is documented for calcineurin inhibitor-related TMA (Izzedine et al., 2014; Rodrigues-Diez et al., 2016; Hošková et al., 2017) and proteasome inhibitors, such as bortezomib, ixazomib and carfilzomib (Lodhi et al., 2015);

This second group has not a clear time onset after drug exposure (classically the TMA occurs after months) and no adding criteria for DITMA diagnosis is needed.

This classification has some limitations. First of all, some drugs can cause TMA both through immune effects and directly. This is described for example for interferon (Kavanagh et al., 2016), oxaliplatin and gemcitabine (Al-Nouri et al., 2015; Saleem et al., 2018). Second, the toxic group comprises different drugs, having heterogeneous biological targets, such as signal transducer, transcription factor or receptors. Moreover, these targets have different distributions above tissues and organs. This target heterogeneity is reflected in clinical DITMA manifestation. In fact, as described above, anti-VEGF and TK inhibitors have tropism for endothelial-podocyte complexes. The deregulation of the podocyte-endothelium complex results in a particular clinical entity described as renal-limited TMA (Izzedine and Perazella, 2015). In contrast to systemic TMA, renal-limited TMA is defined as biopsy-proven renal TMA in the absence of microangiopathic hemolytic anemia and thrombocytopenia. Renal-limited DITMA might be underdiagnosed due to clinical findings limited to the kidney and physicians’ frequent reluctance to perform renal biopsy. This issue becomes even more important in DITMA, considering that TMA renal-limited forms are mainly caused by drugs (28.5%) (Saba et al., 2018). Moreover, if recognized, these forms showed to benefit more from the withdrawal of the causative drug, with a lower relapse risk and better survival rates than the systemic DITMA (Izzedine and Perazella, 2015). These considerations emphasize the importance of considering separately kidney-limited and systemic DITMA forms.

Moreover, some drugs are not able to cause TMA when administered alone but were reported to induce TMA when administered together with other drugs. This includes cytotoxic agents, such as bleomycin and cisplatin (Salhi et al., 2021), and immune checkpoint inhibitors, such as ipilimumab and nivolumab (Lafranchi et al., 2020). In consideration of the criteria listed above for the definition of causative association, we cannot report these drugs as “causative” for TMA. Nevertheless, a synergic effect between endothelial damage, ADAMTS13 deficiency and complement hyperactivation in the development of microangiopathy has already been hypothesized for TMA and demonstrated *in vivo* models (Zheng et al., 2019). There is the possibility that this mechanism is associated with DITMA, but further studies are needed to investigate this hypothesis.

### 3.2 Complement system activation in DITMA

The initial trigger for endothelial cells damage could involve i) platelets, ii) ADAMTS13-activity, iii) endothelial cells, iv) endothelial-podocyte complex. One of the final effectors of endothelial damage is the complement system (Mathern and Heeger, 2015). Mutations of complement proteins such as CFH, C3, CFI, CFB or MCP are clearly associated with development of primary TMA with deregulated activity of the complement system (Noris et al., 2010; Nester et al., 2015). Among secondary TMA forms, the role of the complement is more controversial and difficult to clarify (Bresin et al., 2013; Fremeaux-Bacchi et al., 2013) considering, for example the pan-reactivity of different potential TMA-triggers such as infections (Kobbe et al., 2017), organ and marrow transplantation (Alasfar and Alachkar, 2014), pregnancy (Fakhouri et al., 2010) and, more rarely, drugs (Noris and Remuzzi, 2015). A clear association between complement activation and endothelial damage has not been demonstrated for DITMA. Complement involvement is partially documented in secondary TMAs by high levels of activation products (C3a and sC5b-9), low CFH levels (Farkas et al., 2017), low complement C3 levels (Gilbert et al., 2013), elevated BB fragment (Bhutani et al., 2020; Taghavi et al., 2022), and EC’s deposition of C5b-C9 complex in EC-culture and nephron tissue (Palomo et al., 2019; Blasco et al., 2021). These laboratory/histological data indicate an upregulation of the complement system in TMA.

We performed a literature review in order to understand the extent of the complement activation searching both for decreased serum value of complement proteins and complement deposition in kidney biopsy. More than half of the patients with DITMA who underwent a renal biopsy showed the presence of complement factors at IF staining (38/66; 59%) (Table 2), mainly identified in the capillary wall (24/27; 88%). Among these 38 patients, 25 (66%) have a concomitant evaluation of serum complement factors available which showed normal serum complement levels in 16/25 (64%) patients and decreased complement levels in 9/25 (36%) patients (mainly characterized by with isolated C3 reduction).

Focusing on those patients with decreased serum complement factors, they all had positive IF staining. This concordance may help to identify, based on simple, inexpensive and widely available blood tests, a cluster of patients with an underlying complement hyperactivation. It seems reasonable to treat with anti-complementary therapy, particularly those patients with signs of complement activation.

Different drugs seem to mediate different complement involvement: DITMA cases related to anti-TK (6/7, 95%) and intraocular anti-VEGF (IO anti-VEGF) (5/5, 100%) drugs have a negative complement IF in most of the cases, while DITMA cases secondary to gemcitabine (14/15, 97%) and ramucirumab (11/11, 100%) present complement activation in almost all cases.

These data suggest a variable complement involvement, a likely different susceptibility to anti-complementary drugs and an heterogeneity of the DITMA mechanisms. This will require deeper evaluation of the DITMA pathophysiological mechanism for future reclassifications and drug/disease-based treatment. Nevertheless, several problems still need to be solved to clarify the role of complement in DITMA (as in other secondary TMA forms).

### 3.3 Complement genetic variants in DITMA

The detection of mutations in complement genes of patients with secondary TMA (Bruel et al., 2017; Gaggl et al., 2018; Palma et al., 2021) extended the concept of “deregulated” complement system at the molecular level not only in primary forms of TMA. Some observations highlighted the pivotal role of genetic susceptibility in endothelial damage development; for example, TMA haplotype risks (Esparza-Gordillo et al., 2005; Ding et al., 2017) are more frequent in secondary TMAs (Le Clech et al., 2019) than in healthy samples. Nevertheless, there are suggestions of no genetic involvement (Le Clech et al., 2019; Fakhouri and Frémeaux-Bacchi, 2021) in the genesis of endothelial damage, in favor of single (40% of cases) or multiple (60% of cases) environmental triggers (Bayer et al., 2019).

Genetic variants in complement genes are found in less than 10% of DITMA cases (Cavero et al., 2017; Palma et al., 2021), and only a small number of case reports is reported on the detection of potential causative complement variants in DITMA (Gilbert et al., 2013; Atallah-Yunes and Soe, 2018).

To the best of our knowledge, in literature, the total number of DITMA patients with available genetic panels for the screening of complement genes is 122 (Table 3). The whole exome sequencing was not carried out in any case. Pathogenic variants (4–5 class in ACMG classification) were reported in only two patients (2/122, 1.7%) and variants of unknown significance (VOUS) or class 3 in ACMG classification were found in 12/98 (12.2%) also considering CFHR1-3 deletions. However, as suggested by recent data (Le Clech et al., 2019; Fakhouri and Frémeaux-Bacchi, 2021), those variants are not clearly associated with TMA susceptibility and should not be considered clear genetic risk factors for TMA. This lowers the total number of patients with VOUS to 9/98 (9.1%). In the general population, VOUS in complement genes (mainly CFH and CFHR) are reported in 5%–10% of healthy controls (Le Clech et al., 2019; Fakhouri and Frémeaux-Bacchi, 2021). The percentage of VOUS in DITMA cases is not significantly different from that of the general population. Taking these data into account, the role of pathogenic variants in complement genes in DITMA is probably limited to a few exceptional cases (2/122, 1.7% of patients in literature with likely pathogenic or pathogenic variant), differently from what happened in aHUS, malignant hypertension-associated TMA (Zhang et al., 2020) and pregnancy-related TMA (Frémeaux-Bacchi et al., 2019). In support of this, the DITMAs showed a very low relapse rate (Le Clech et al., 2019).

The absence of genetic abnormalities in most patients does not exclude participation of complement in the pathogenesis of DITMA and other secondary TMAs. Complement dysregulation is not equivalent to complement activation. In fact, the activated procoagulant and proinflammatory endothelial cell phenotype characteristic of DITMA can induce a non-specific activation of complement, which in turn can promote and aggravate TMA (Duineveld and Wetzels, 2019; Frémeaux-Bacchi et al., 2019).

## 4 Clinical presentation of DITMA

Diagnosis of TMA has historically been dependent on clinical parameters at presentation, with a classic/distinct clinical triad: microangiopathic hemolytic anemia (increased serum lactate dehydrogenase, haptoglobin consumption and schistocytes in the blood smear), thrombocytopenia, and renal injury or failure. Not all of these items are needed simultaneously to confirm the presence of TMA (Brocklebank et al., 2018). In fact, it is increasingly recognized that TMA may present with hypertension and/or renal function impairment with no or mild thrombocytopenia and/or no microangiopathic hemolytic anemia, ranging from subclinical laboratory abnormalities to full-blown TMA (Brocklebank et al., 2018; Estrada et al., 2019; Valério et al., 2021). In particular, also some DITMA cases have a subacute presentation with mild or only relative thrombocytopenia (>25% reduction in platelet count from baseline) and gradual deterioration of renal function, and the identification of schistocytes by peripheral smear may not be documented in all cases (Eremina et al., 2008).

The maximum expression of this atypical/subclinical TMA form is the renal-limited TMA, the majority of whose cases are caused by drugs (28.5%) (Saba et al., 2018). The absence of usual systemic findings makes the TMA diagnosis difficult. Renal-limited TMA at renal biopsy shows fibrin thrombi, glomerular mesangiolysis, subendothelial space widening with endothelial cells detaching from the glomerular basement membrane, endothelial swelling in glomeruli, arterioles and interlobular arteries with fibrinoid necrosis (Fakhouri et al., 2017; Goodship et al., 2017). Multiple drugs have been described in association with renal-limited TMA: anti-VEGF, TK inhibitors and proteasome inhibitors (Estrada et al., 2019; Valério et al., 2021). Renal-limited DITMA showed to benefit greatly from the withdrawal of the causative drug, with a lower relapse risk and better survival rates compared to systemic DITMA (Izzedine and Perazella, 2015).

Once the TMA is diagnosed, the focus becomes identifying the underlying etiology (Brocklebank et al., 2018). In particular, DITMA is indistinguishable from other TMA forms. In general, the clinical presentation of immune-mediated DITMA is characterized by an acute onset usually triggered by first contact with the drug, on the other hand DITMA caused by direct dose-dependent drug toxicity are characterized by acute or subacute onset together with systemic features on initial or prolonged exposure to the drug (George and Nester, 2014). In our review, we have noticed that some drugs may lead to a more distinctive clinical scenario. For instance, IFN-related DITMA is generally characterized by a fulminant presentation with classical TMA clinical manifestations accompanied by severe headache and severe/malignant hypertension in most of the patients, or may alternatively describe progressive worsening of hypertension, proteinuria, and headache (Hunt et al., 2014; Dauvergne et al., 2021). A new or exacerbated hypertension was a prominent feature also in gemcitabine-induced DITMA, together with signs/symptoms of severe fluid overload (rarely resembling capillary leak syndrome) (Walter et al., 2002; Humphreys et al., 2005). Although ticlopidine is not listed among causative TMA drugs (due to a time >45 days between drug exposure and TMA onset), a clinical scenario similar to ticlopidine-associated TTP has been described (Zakarija et al., 2009; Bennett et al., 2013), characterized by neurological symptoms and decreased ADAMTS-13 activity.

In conclusion, awareness on the wide spectrum of DITMA clinical manifestation may facilitate its recognition and the causative drug’s withdrawal before serious complications or death occurs.

## 5 Treatment of DITMA

Typically, proper management of DITMA mainly involves withdrawal of the suspected causative drug and supportive care. Resolution or improvement of TMA is observed after the drug is stopped or dose reduced; however, discontinuation alone is often not enough to lead to clinical recovery and some degree of kidney injury can persist. In these patients, especially in case of advanced kidney disease, other therapies need to be considered. The poor understanding of the underlying etiopathogenetic mechanisms of DITMA and the absence of clinical trials on this topic are reflected in treatment guidelines not supported by strong scientific evidence.

### 5.1 Therapeutic plasma exchange and rituximab in the treatment of DITMA

Therapeutic plasma exchange (TPE) (Zbaras et al., 2013; Cepeda et al., 2018) and rituximab (RTX) (Gourley et al., 2010; Murugapandian et al., 2015) have been utilized among patients not responding to causative drug withdrawal. The treatment with TPE or RTX should be considered in immune-mediated DITMA, as described in association with quinine, gemcitabine, cisplatin and clopidogrel (Jacob et al., 2012; Padmanabhan et al., 2019). Both TPE and RTX lead to removal of drug dependent-antibodies, the first one *via* mechanical removal of autoantibodies (Nguyen et al., 2019; Jung et al., 2022) and the ladder permitting apoptosis of autoantibody producing cells as in other forms of TTP (Au et al., 2007; Jestin et al., 2018; Jung et al., 2022). TPE has been applied to immune -mediated DITMA based on the former positive experience with TTP, although there is no data on TPE efficacy or TPE superiority compared with other techniques (such as plasma infusion). Retrospective and prospective studies on large DITMA cohorts who underwent RTX therapy are missing. In relation to TPE, to the best of our knowledge, only retrospective cohort studies are reported, mainly for gemcitabine, clopidogrel and quinine. In gemcitabine-related TMA, patients treated with TPE showed a remission rate and prognosis similar to patients treated with only drug suspension, although in presence of more severe kidney and hematological manifestations at baseline (Gore et al., 2009; Daviet et al., 2019). In clopidogrel-associated TMA, similar remission rates are reported in both TPE and non TPE-treated groups (Bennett et al., 2007; Jacob et al., 2012). In quinine-associated TMA, comparative data on different remission rates between TPE and non TPE-treated groups are not reported because all recruited patients were treated with plasma-exchange (Page et al., 2017). Retrospective studies are also reported for TPE in ticlopidine-associated TMA (Bennett et al., 2007; Jacob et al., 2012). Although there are numerous concerns on the association between ticlopidine and TMA (due to temporal criteria), hematological and renal remission rates in TPE-treated patients are significantly better compared to non TPE-treated patients (Jacob et al., 2012; Bennett et al., 2013), supporting the possible involvement of drug-induced antibodies (with reduced anti-ADAMTS13 activity) in determining the ticlopidine-induced clinical scenario similar to TTP.

The utility of TPE in other etiologies of DITMA is debatable. TPE would be expected to be effective in immune-mediated DITMA, whereas it would not be with those medications in which direct endothelial injury occurs (Winters, 2017). However, drugs described to have direct endothelial toxicity induce release of large amounts of von Willebrand factor multimers and concomitant activation of the coagulation cascade. The theoretical concept of TPE in DITMA combines two main aspects: removal of harmful circulating molecules released during the endothelial damage (such as vWF multimers, proinflammatory cytokines and permeability factors) that directly contribute to the underlying etiopathogenetic mechanism, and replacement of physiologic plasma proteins (e.g., activated protein C, antithrombin, tissue factor pathway inhibitor, vWF cleaving proteases, VEGF, complement regulator factors) to ultimately restore hemostasis. In patients with DITMA, in association with TPE, corticosteroids (intravenous and/or per os) are used, but data on treatment efficacy are missing. Considering the underlying etiopathogenetic mechanism, their use could find application in immune-mediated DITMA.

Based on data currently available in the literature and considering the limited evidence, we suggest that the use of TPE and RTX in DITMA must be weighed and limited to a specific subset of patients with suspect of immune-mediate DITMA, in particular in the case of a possible association with ticlopidine, and in non-responsive to drug withdrawal (Izzedine and Perazella, 2015; Schwartz et al., 2016; Brocklebank et al., 2018; Padmanabhan et al., 2019). Moreover, according to consensus opinion (Palma et al., 2021), regardless of whether a toxic or immune-mediated form, a trial of TPE is recommended in all patients with a suspected diagnosis of severe DITMA, in addition to suspected-causative drug suspension. During TPE, platelet count, haemolysis lab tests and renal function should be carefully monitored. In summary, other studies are needed to confirm a potential role of TFE and RTX in DITMA.

### 5.2 Complement inhibitor in DITMA treatment

Given the role played by complement system activation, anti-complement drugs have been proposed as a therapeutic option in DITMA. Eculizumab is a monoclonal drug that inhibits C5-factor, thus not permitting the formation of the Complement Membrane Attack Complex, avoiding, the facto, cell lysis and death.

Eculizumab is approved for the treatment of Paroxysmal Nocturnal Hemoglobinuria and aHUS, although it is widely adopted for patients with severe secondary TMAs (Caravaca-Fontan and Praga, 2019), even if its efficacy has not been demonstrated in validated clinical trials. The overactivation or deregulation of complement system is not proven in DITMA patients and, consequently, indications for eculizumab treatment are limited. Some Authors (Cavero et al., 2017; Caravaca-Fontan and Praga, 2019) suggest to use the anti-complement therapy only in case of lack of improvement of hematological parameters and/or renal function recovery after causative drug discontinuation. This approach lacks supporting evidence, which is currently based on case reports and case series. Other Authors (Duineveld and Wetzels, 2019)do not suggest the use of eculizumab in secondary DITMA cases, relying on data indicating that renal outcome was not significantly altered by the use of the complement inhibitor. Several case report and little case series suggested the effectiveness of C5-inhibitor in DITMA in terms of normalization of hematological parameters and, in some cases, partial or complete recovery of kidney function (Allinovi et al., 2017; Cavero et al., 2017; Caravaca-Fontan and Praga, 2019; Grall et al., 2021). In particular, eculizumab proved its efficacy in three out of 9 (33.3%) patients who resolved the IFN-related TMA without further complications, and in four out of six patients who needed dialysis treatment at DITMA onset and discontinued dialysis after a mean of 2 months of eculizumab treatment (Allinovi et al., 2021). Moreover (Grall et al., 2021), reported a significantly better kidney response (83% vs. 64% of complete/partial recovery) and kidney outcome (eGFR 45 vs. 33 ml/min/1.73) in 13 patients with gemcitabine-induced TMA treated with anti-complement therapy. However, conflicting results were reported by previous articles gemcitabine-induced TMA treated with C5-inhibitor (Al Ustwani et al., 2014; Daviet et al., 2019). Data on large DITMA cohorts and on other causative DITMA drugs are not available.

As we reported in the previous paragraph, decreased C3 fraction in blood and/or deposition of C3 fraction in kidney biopsy may be considered as “red flags” of an overactivation of complement system, on which the choice to start a treatment with a complement inhibitor should be focused.

In this review, considering that DITMAs rarely conceal primitive TMAs (2/122, 1.6%), we support the flow-chart of TMA treatment previously proposed (Brocklebank et al., 2018; Caravaca-Fontan and Praga, 2019) in which the removal of the trigger should be pursued in suspected secondary TMAs (i.e. withdrawal of the offending drug in DITMA). The use of eculizumab in DITMA is suggested in severe TMA cases, and when the drug withdrawal and 3–5 TPE sessions do not associate with an improvement in renal function and hematological parameters. In these cases, complement-mediated TMA may need to be considered as an alternative diagnosis. In fact, it is difficult to discriminate the etiology (primary vs. secondary) in the acute phase of TMA due to different reasons:

- Primary TMA is suspected based on the absence of “secondary causative factors” such as drugs, cancer, infection or pregnancy. However, primary TMA diagnosis is excluded only by negative genetic analysis, and “secondary causative factors” can frequently trigger primary TMA cases. Moreover, timing for the genetic analysis of complement factors generally takes 1–6 months, an aspect that does not fit with the acuteness and severity of TMA;
- In DITMA, the causative relationship between drugs and TMA is usually not clear and more than one triggering factor is frequently present.

In conclusion, the efficacy of eculizumab treatment in DITMA needs to be clarified with further prospective studies, with homogeneous timing of eculizumab infusion and selection of patients with similar clinical and prognostic features.

## 6 Conclusion

DITMA is one of the most common forms of TMA. It is a potentially life-threatening disease. The mechanisms of drug-induced endothelial damage are heterogenous and not completely understood. In terms of pathological mechanisms, genetic variants in complement genes are found very rarely in DITMA patients, while an acquired (both systemic or renal) complement hyperactivation was documented in more than a half of the DITMA patients. The potentially rapid reversal of the pathological signs in DITMA patients underlines the importance of early suspicion, recognition and discontinuation of the potentially offending drug, in order to reduce organ dysfunction. In severe DITMA cases not responsive to drug withdrawal, the use of TPE, RTX or eculizumab can be used as rescue therapy. There are currently no prospective studies supporting the efficacy of these treatments in DITMA.

Further studies are needed to understand mechanisms of drugs-related damage, the role of complement activation and the efficacy of TPE, RTX and anti-complement therapy in DITMA.
