# Supplementary material for: Drug-induced thrombotic microangiopathy: An updated review of causative drugs, pathophysiology, and management
Source: Front Pharmacol. 2023 Jan 9;13:1088031. doi: 10.3389/fphar.2022.1088031 (PMC9868185; doi:10.3389/fphar.2022.1088031)
Supplement: Supplementary file 1 [file Table1.DOCX]

**Supplementary material**

**Table S1. New drugs with a possible or definite association with TMA.**

| Drug | Author | Score＊ |
| --- | --- | --- |
| Alemtuzumab | Liou AA, 2019 | 1 |
| Certolizumab pegol | Baysal M, 2017 | 1 |
| Hydroxychoroquine | Arıkan F, 2020 | 2 |
| Leflunomide | Shields MD, 2021 | 1 |
| Lenvatinib | Nakashima S, 2022 | 1 |
| Levofloxacin | Kazi S, 2018 | 1 |
| Lomustine | Alrohaibani A, 2020 | 1 |
| Moxetumomab Pasudotox | Wayne AS, 2017 | 1 |
| Nintedanib | Hasegawa M, 2021 | 1 |
| Onasemnogene abeparvovec | Chand DH, 2021 | 1 |
| Pazopanib | Syed U, 2018 | 2 |
| Pegylated liposomal doxorubicin | Rodriguez-Ramirez S, 2022 | 1 |
| Ramucirumab | Yamada et al,2019 | 1 |
| Regorafenib | Yin Q,2022 | 1 |
| Rifampicin | Hamad H,2020 | 2 |
| Valacyclovir | Buckhari S, 2020 | 2 |

＊The association score is based on the same criteria reported in Al-Nouri et al, 2015 and Saleem et al.

**Supplementary citation in Table 1 [S1-S17]**

S1. [Saleem R, Reese JA, George JN (2018) Drug-induced thrombotic microangiopathy: An updated systematic review, 2014-2018. American Journal of Hematology 93:E241–E243](http://paperpile.com/b/VKXZSx/cXAQ)

S2. [Liou AA, Skiver BM, Yates E, et al (2019) Acute Thrombotic Microangiopathy and Cortical Necrosis Following Administration of Alemtuzumab: A Case Report. Am J Kidney Dis 73:615–619](http://paperpile.com/b/VKXZSx/prd5)

S3. [Baysal M, Ümit EG, Sarıtaş F, et al (2018) Drug Induced Thrombotic Microangiopathy with Certolizumab Pegol. Balkan Med J 35:398–399](http://paperpile.com/b/VKXZSx/e5zM)

S4. [Arıkan F, Yıldız Y, Ercan T, et al (2020) Hydroxychloroquine-Associated Thrombotic Thrombocytopenic Purpura. Turk J Haematol 37:302–304](http://paperpile.com/b/VKXZSx/pS0Q)

S5. [Shields MD, Skelton WP 4th, Laber DA, et al (2021) A Novel Case of Leflunomide-Induced Thrombotic Thrombocytopenic Purpura. J Hematol 10:139–142](http://paperpile.com/b/VKXZSx/aZBj)

S6. [Nakashima S, Sekine A, Sawa N, et al (2022) A Case of Thrombotic Microangiopathy, Podocytopathy, and Damage to the Renal Tubules with Severe Proteinuria and Acute Renal Dysfunction Induced by Lenvatinib. Intern Med. https://doi.org/](http://paperpile.com/b/VKXZSx/wktA)[10.2169/internalmedicine.8365-21](http://dx.doi.org/10.2169/internalmedicine.8365-21)

S7. [Kazi S, Preston GC (2018) Drug induced thrombotic microangiopathy caused by levofloxacin. J R Coll Physicians Edinb 48:127–129](http://paperpile.com/b/VKXZSx/evYQ)

S8. [Alrohaibani A, Swetnam LA, Andeen NK (2020) Microangiopathy, lomustine, and karyomegalic mesangial cells. Kidney Int 98:517](http://paperpile.com/b/VKXZSx/hiDt)

S9. [Wayne AS, Shah NN, Bhojwani D, et al (2017) Phase 1 study of the anti-CD22 immunotoxin moxetumomab pasudotox for childhood acute lymphoblastic leukemia. Blood 130:1620–1627](http://paperpile.com/b/VKXZSx/Q5Le)

S10. [Hasegawa M, Uehara A, Suzuki T, et al (2020) Nintedanib-induced glomerular microangiopathy: a case report. CEN Case Rep 9:295–300](http://paperpile.com/b/VKXZSx/yXF2)

S11. [Chand DH, Zaidman C, Arya K, et al (2021) Thrombotic Microangiopathy Following Onasemnogene Abeparvovec for Spinal Muscular Atrophy: A Case Series. J Pediatr 231:265–268](http://paperpile.com/b/VKXZSx/JSBN)

S12. [Syed U, Wahlberg KJ, Douce DR, Sprague JR (2018) Thrombotic Thrombocytopenic Purpura Associated with Pazopanib. Case Rep Hematol 2018:4327904](http://paperpile.com/b/VKXZSx/bspN)

S13. [Rodriguez-Ramirez S, Yau K, Kitchlu A, et al (2022) Pegylated Liposomal Doxorubicin and Kidney-Limited Thrombotic Microangiopathy in a Kidney Transplant Recipient: A Case Report. Kidney Med 4:100461](http://paperpile.com/b/VKXZSx/akps)

S14. [Yamada R, Okawa T, Matsuo K, et al (2019) Renal-limited thrombotic microangiopathy after switching from bevacizumab to ramucirumab: a case report. BMC Nephrol 20:14](http://paperpile.com/b/VKXZSx/a7uK)

S15. [Yin Q, Guo N, Zhou X, et al (2022) Regorafenib-induced renal-limited thrombotic microangiopathy: a case report and review of literatures. BMC Nephrol 23:112](http://paperpile.com/b/VKXZSx/5dgq)

S16. [Hamad H, Sahu KK, Dunn S, et al (2020) Rifampin Induced Thrombotic Thrombocytopenic Purpura. Indian J Hematol Blood Transfus 36:575–577](http://paperpile.com/b/VKXZSx/z32P)

S17. [Bukhari S, Aslam HM, Awwal TA, et al (2020) Valacyclovir-Induced Thrombotic Thrombocytopenic Purpura. Cureus](http://paperpile.com/b/VKXZSx/0evr)

**Supplementary citation Table 2 [S18-S53]**

S18. [Cavoli GL, Li Cavoli G, Bono L, et al (2011) Renal thrombotic microangiopathy induced by -interferon. Clinical Kidney Journal 4:80–80](http://paperpile.com/b/8JQRmA/o7y3)

S19. [Broughton A, Cosyns J-P, Jadoul M (2011) Thrombotic microangiopathy induced by long-term interferon-β therapy for multiple sclerosis: a case report. Clinical Nephrology 76:396–400](http://paperpile.com/b/8JQRmA/6fX5)

S20. [Olea T, Díaz-Mancebo R, Picazo M-L, et al (2012) Thrombotic microangiopathy associated with use of interferon-beta. Int J Nephrol Renovasc Dis 5:97–100](http://paperpile.com/b/8JQRmA/0Tl5)

S21. [Nerrant E, Charif M, Ramay A-S, et al (2013) Hemolytic uremic syndrome: an unusual complication of interferon-β treatment in a MS patient. Journal of Neurology 260:1915–1916](http://paperpile.com/b/8JQRmA/GPlw)

S22. [Piccoli GB, Capobianco M, Vigotti FN, et al (2016) The Case | The young philosopher with multiple sclerosis and proteinuria. Kidney Int 89:961–963](http://paperpile.com/b/8JQRmA/9tji)

S23. [Allinovi M, Cirami CL, Caroti L, et al (2017) Thrombotic microangiopathy induced by interferon beta in patients with multiple sclerosis: three cases treated with eculizumab. Clinical Kidney Journal 10:625–631](http://paperpile.com/b/8JQRmA/WYMU)

S24. [Gianassi I, Allinovi M, Caroti L, Cirami LC, et al (2019) Broad spectrum of interferon-related nephropathies-glomerulonephritis, systemic lupus erythematosus-like syndrome and thrombotic microangiopathy: A case report and review of literature. World Journal of Nephrology 8:109–117](http://paperpile.com/b/8JQRmA/VdKV)

S25. Parisi M, Manni A, Caputo F, Trojano M, Paolicelli D (2020) A case report of late-onset atypical Hemolytic Uremic Syndrome during interferon beta in multiple sclerosis: Open issues in literature review. Brain Behav 00:e01930. https://doi.org/10.1002/brb3.1930

S26. [Allinovi M, Bellinvia A, Pesce F, et al (2021) Safety and Efficacy of Eculizumab Therapy in Multiple Sclerosis: A Case Series. Brain Sciences 11:1341](http://paperpile.com/b/rYgL9S/pzD4)

S27. Murugapandian S, Bijin B, Mansour I, Daheshpour S, Pillai B, G, Thajudeen B, Salahudeen A, K (2015) Improvement in Gemcitabine-Induced Thrombotic Microangiopathy with Rituximab in a Patient with Ovarian Cancer: Mechanistic Considerations. Case Rep Nephrol Dial 5:160-167. doi: 10.1159/000435807

S28. [Krishnappa V, Gupta M, Shah H, et al (2018) The use of eculizumab in gemcitabine induced thrombotic microangiopathy. BMC Nephrology 19](http://paperpile.com/b/8JQRmA/stNn)

S29. [Burns ST, Damon L, Akagi N, et al (2020) Rapid Improvement in Gemcitabine-associated Thrombotic Microangiopathy After a Single Dose of Eculizumab: Case Report and Review of the Literature. Anticancer Res 40:3995–4000](http://paperpile.com/b/8JQRmA/GhE6)

S30. [Grall M, Daviet F, Chiche NJ, et al (2021) Eculizumab in gemcitabine-induced thrombotic microangiopathy: experience of the French thrombotic microangiopathies reference centre. BMC Nephrology 22](http://paperpile.com/b/8JQRmA/p5ff)

S31. [Etta P, Gowrishankar S (2020) Clopidogrel induced thrombotic microangiopathy successfully treated with conservative approach. Indian Journal of Nephrology 30:209](http://paperpile.com/b/8JQRmA/jKJ9)

S32. [Mizuno S, Kitayama C, Sanada S, Sato T (2021) Bortezomib-induced glomerular microangiopathy complicated with monoclonal immunoglobulin deposition disease. CEN Case Reports 10:537–542](http://paperpile.com/b/8JQRmA/VQzj)

S33. [Hobeika L, Self SE, Velez JCQ (2014) Renal thrombotic microangiopathy and podocytopathy associated with the use of carfilzomib in a patient with multiple myeloma. BMC Nephrology 15](http://paperpile.com/b/8JQRmA/wqHz)

S34. Yamada R, Okawa T, Matsuo K, Suzuki M, Mori N, Mori K. Renal-limited thrombotic microangiopathy after switching from bevacizumab to ramucirumab: a case report. BMC Nephrol. 2019 Jan 11;20(1):14. doi: 10.1186/s12882-018-1194-9.

S35. [Ozawa M, Komatsuda A, Ohtani H, et al (2017) Long-term prognosis of AL and AA renal amyloidosis: a Japanese single-center experience. Clinical and Experimental Nephrology 21:212–227](http://paperpile.com/b/8JQRmA/Orni)

S36. [Nakano Y, Kumagai J, Nagahama K, Fujisawa H (2021) A case of ramucirumab-induced renal failure with nephrotic-range proteinuria and its pathological findings. BMJ Case Rep 14.: https://doi.org/](http://paperpile.com/b/8JQRmA/SuTt)[10.1136/bcr-2020-239603](http://dx.doi.org/10.1136/bcr-2020-239603)

S37. [Pellé G, Shweke N, Van Huyen J-PD, et al (2011) Systemic and Kidney Toxicity of Intraocular Administration of Vascular Endothelial Growth Factor Inhibitors. American Journal of Kidney Diseases 57:756–759](http://paperpile.com/b/8JQRmA/aHR6)

S38. [Touzani F, Geers C, Pozdzik A (2019) Intravitreal Injection of Anti-VEGF Antibody Induces Glomerular Endothelial Cells Injury. Case Reports in Nephrology 2019:1–4](http://paperpile.com/b/8JQRmA/jzuv)

S39. [Hanna RM, Tran N-T, Patel SS, et al (2020) Thrombotic Microangiopathy and Acute Kidney Injury Induced After Intravitreal Injection of Vascular Endothelial Growth Factor Inhibitors VEGF Blockade-Related TMA After Intravitreal Use. Front Med 7:579603](http://paperpile.com/b/8JQRmA/9Yd0)

S40. Miller AJ, Chang A, Cunningham PN. Chronic Microangiopathy Due to DCR-MYC, a Myc-Targeted Short Interfering RNA. Am J Kidney Dis. 2020 Apr;75(4):513-516. doi: 10.1053/j.ajkd.2019.09.011.

S41. [Eremina V, Jefferson JA, Kowalewska J, et al (2008) VEGF inhibition and renal thrombotic microangiopathy. N Engl J Med 358:1129–1136](http://paperpile.com/b/8JQRmA/WLDF)

S42. [Roncone D, Satoskar A, Nadasdy T, et al (2007) Proteinuria in a patient receiving anti-VEGF therapy for metastatic renal cell carcinoma. Nat Clin Pract Nephrol 3:287–293](http://paperpile.com/b/8JQRmA/eDb0)

S43. [Toriu N, Sekine A, Mizuno H, et al (2019) Renal-Limited Thrombotic Microangiopathy due to Bevacizumab Therapy for Metastatic Colorectal Cancer: A Case Report. Case Reports in Oncology 12:391–400](http://paperpile.com/b/8JQRmA/VZYi)

S44. [Morimoto M, Arai T, Matsuura M, Ono Y (2021) Bevacizumab-associated glomerular microangiopathy that occurred after postoperative chemotherapy for ovarian cancer. CEN Case Reports 10:6–11](http://paperpile.com/b/8JQRmA/uDoA)

S45. [Yılmaz S, Özçakar ZB, Taktak A, et al (2016) Anti-VEGF-related thrombotic microangiopathy in a child presenting with nephrotic syndrome. Pediatr Nephrol 31:1029–1032](http://paperpile.com/b/8JQRmA/7xAQ)

S46. [Morita N, Ozaki T, Yokota S, et al (2022) Focal segmental glomerulosclerosis and concurrent glomerular microangiopathy after long-term imatinib administration. CEN Case Reports 11:134–140](http://paperpile.com/b/8JQRmA/tfcG)

S47. [Patel TV, Morgan JA, Demetri GD, et al (2008) A preeclampsia-like syndrome characterized by reversible hypertension and proteinuria induced by the multitargeted kinase inhibitors sunitinib and sorafenib. J Natl Cancer Inst 100:282–284](http://paperpile.com/b/8JQRmA/KuWS)

S48. [Bollée G, Patey N, Cazajous G, et al (2009) Thrombotic microangiopathy secondary to VEGF pathway inhibition by sunitinib. Nephrol Dial Transplant 24:682–685](http://paperpile.com/b/8JQRmA/FUMA)

S49. [Jha PK, Vankalakunti M, Siddini V, et al (2013) Sunitinib induced nephrotic syndrome and thrombotic microangiopathy. Indian J Nephrol 23:67–70](http://paperpile.com/b/8JQRmA/EUwH)

S50. [Nieto-Ríos JF, García-Prada CA, Aristizabal-Alzate A, et al (2021) Síndrome nefrótico como manifestación de microangiopatía trombótica secundaria al uso crónico de sunitinib. Nefrología](http://paperpile.com/b/8JQRmA/oTtD)

S51. [Hyogo Y, Kiyota N, Otsuki N, et al (2018) Thrombotic Microangiopathy with Severe Proteinuria Induced by Lenvatinib for Radioactive Iodine-Refractory Papillary Thyroid Carcinoma. Case Reports in Oncology 11:735–741](http://paperpile.com/b/8JQRmA/639C)

S52. [Hasegawa M, Uehara A, Suzuki T, et al (2020) Nintedanib-induced glomerular microangiopathy: a case report. CEN Case Rep 9:295–300](http://paperpile.com/b/8JQRmA/O6rz)

S53. [Maruyama K, Nakagawa N, Suzuki A, et al (2018) Pazopanib-induced Endothelial Injury with Podocyte Changes. Internal Medicine 57:987–991](http://paperpile.com/b/8JQRmA/S5Rv)

**Supplementary citation Table 3 [S54-S66]**

S54. [Hunt D, Kavanagh D, Drummond I, et al (2014) Thrombotic microangiopathy associated with interferon beta. N Engl J Med 370:1270–1271](http://paperpile.com/b/rYgL9S/EEz8)

S55. [Allinovi M, Bellinvia A, Pesce F, et al (2021) Safety and Efficacy of Eculizumab Therapy in Multiple Sclerosis: A Case Series. Brain Sciences 11:1341](http://paperpile.com/b/rYgL9S/pzD4)

S56. [Dauvergne M, Buob D, Rafat C, et al (2021) Renal diseases secondary to interferon-β treatment: a multicentre clinico-pathological study and systematic literature review. Clinical Kidney Journal 14:2563–2572](http://paperpile.com/b/rYgL9S/thq6)

S57. [Le Clech A, Simon-Tillaux N, et al (2019) Atypical and secondary hemolytic uremic syndromes have a distinct presentation and no common genetic risk factors. Kidney International 95:1443–1452](http://paperpile.com/b/rYgL9S/ROeb)

S58. [Schulte-Kemna L, Reister B, Bettac L, et al (2020) Eculizumab in chemotherapy-induced thrombotic microangiopathy. Clinical Nephrology – Case Studies 8:25–3](http://paperpile.com/b/rYgL9S/AYEc)2

S59. [Grall M, Daviet F, Chiche NJ, et al (2021) Eculizumab in gemcitabine-induced thrombotic microangiopathy: experience of the French thrombotic microangiopathies reference centre. BMC Nephrology 22](http://paperpile.com/b/rYgL9S/afHe)

S60. [Page EE, Little DJ, Vesely SK, George JN (2017) Quinine-Induced Thrombotic Microangiopathy: A Report of 19 Patients. American Journal of Kidney Diseases 70:686–695](http://paperpile.com/b/rYgL9S/qohX)

S61. [Izzedine H, Mangier M, Ory V, et al (2014) Expression patterns of RelA and c-mip are associated with different glomerular diseases following anti-VEGF therapy. Kidney Int 85:457–470](http://paperpile.com/b/rYgL9S/Cxeh)

S62. [Cavero T, Rabasco C, López A, et al (2017) Eculizumab in secondary atypical haemolytic uraemic syndrome. Nephrol Dial Transplant 32:466–474](http://paperpile.com/b/1VaD0H/wk2N)

S63. [Yui JC, Van Keer J, Weiss BM, et al (2016) Proteasome inhibitor associated thrombotic microangiopathy. Am J Hematol 91:E348–52](http://paperpile.com/b/rYgL9S/UTVY)

S64. [Portuguese AJ, Lipe B (2018) Carfilzomib-induced aHUS responds to early eculizumab and may be associated with heterozygous CFHR3-CFHR1 deletion. Blood Adv 2:3443–3446](http://paperpile.com/b/rYgL9S/85uu)

S65. [Gavriilaki, Dalampira, Theodorakakou, et al (2022) Genetic and Functional Evidence of Complement Dysregulation in Multiple Myeloma Patients with Carfilzomib-Induced Thrombotic Microangiopathy, J Clin Rheumatol](http://paperpile.com/b/StWetV/qyHk)

S66. [Chapin J, Eyler S, Smith R, et al (2013) Complement factor H mutations are present in ADAMTS13-deficient, ticlopidine-associated thrombotic microangiopathies. Blood 121:4012–4013](http://paperpile.com/b/rYgL9S/ZAqx)
